# Flexible Goal Adjustment Predicts Well‐Being Before and After Retirement

**DOI:** 10.1155/jare/2950011

**Published:** 2026-01-28

**Authors:** Lisa M. Viegas, Christina Bermeitinger, Werner Greve

**Affiliations:** ^1^ Institute of Psychology, University of Hildesheim, Hildesheim, Germany, uni-hildesheim.de

**Keywords:** critical life change, goal adjustment, retirement outcomes, self-regulation

## Abstract

**Background and Objectives:**

Retirement represents a critical life change and is accompanied by the blocking of professional goals. We assume that the individual capability to adapt goals to changed conditions has a palliative effect on well‐being. The aim of the present study was to assess interindividual differences in goal adjustment and to analyse the predictive value of these goal adjustments for personal well‐being.

**Research Design and Methods:**

206 pastors participated in a self‐report online study. We assessed their current working status, individual preparedness for flexible goal adjustment (FGA), and the current importance of private and professional goals. Furthermore, their well‐being and self‐esteem were measured.

**Results:**

Our data showed that retired individuals rated professional, but not private, goals as less important than working individuals. Additionally, FGA predicted self‐esteem and well‐being for both working and retired individuals.

**Discussion and Implications:**

While there was no correlation between FGA and the importance of professional goals, the results still indicate that goal adjustment can help maintain well‐being in retirement.

## 1. Introduction

### 1.1. Maintaining Well‐Being in Retirement: The Role of Goal Adjustment

Maintaining well‐being and quality of life in old age is a central challenge for people when losses accumulate, threats increase, individual resources decrease, and cultural compensation options become less efficient [1–5]. At the same time, numerous findings show that most older people succeed: on average, well‐being remains stable into old age (e.g., [6–8]; but also see [9], for international differences in that trend). Several models of developmental regulation have been proposed, which aim to explain how well‐being is maintained. A common feature of these models is the focus on the protective function of goal adjustment ([10–12]; for a comparison of these models, see [13]) and explaining how these processes evolve with time in the face of ubiquitous age‐correlated losses [13]. In this tradition, a number of studies indicates that willingness to let go of unattainable goals is associated with higher well‐being and better mental health (e.g., [10, 12, 14–16]), as well as with fewer negative consequences of a goal blockage, such as depressive symptoms, anxiety, or perceived stress [17, 18]. This suggests that the impact of a confrontation with developmental transitions that involve a blockage of relevant goals can be mitigated if the person can reduce the subjective importance of the now blocked goals. Although these theories were originally developed with a particular focus on older adulthood, there are still too few studies that specifically examine concrete, profound developmental transitions in old age.

### 1.2. Retirement as a Normative Critical Life Event: The Challenge of Change

Findings suggest that the transition to retirement, although it can be anticipated as a normative critical life event, is often experienced as a challenge [19]. Of course, perceptions of this transition vary between individuals, and of course each person will associate retirement with more than one evaluation (loss (of income or status) and gain (of leisure time and leeway for social engagement) and relief (of burden of responsibility) and concern (search for new sources of meaning), etc.), but for almost all people who experience it as an event (as the scheduled end of regular professional activity), retirement will be a developmental transition that profoundly changes the constellation of their lives. Accordingly, the individual’s transition into retirement entails processes through which retirees get used to the changed aspects of life that result from the work–retirement transition and seek to achieve psychological comfort with their retirement life [20]. However, the question whether retirees actually achieve psychological comfort has been discussed for decades. Opposing theoretical approaches (from disengagement theory, [21], to successful aging, [22, 23]) and seemingly divergent findings (e.g., [19, 24, 25]) indicate that a simple answer is implausible. Rather, the findings suggest that not retirement per se predicts individual responses to this transition, but that moderating factors alter, enforce, mitigate, or buffer its consequences. Accordingly, the focus of investigation has shifted from the mere description of consequences of retirement (e.g., with respect to life satisfaction or well‐being) towards contextual and individual factors influencing and qualifying the impact of retirement on well‐being [19]. Research here has often focused on contextual factors (e.g., financial resources, [26]; marital status, [27]; or partners’ life satisfaction, [28]). However, the heterogeneous findings speak in favour of looking for individual psychological moderators as well. For instance, Hansson et al. [29] found that individual resources (such as self‐esteem) predict life satisfaction. In the search for theoretically promising individual moderators of the relationship between transition to retirement and quality of life, it is worth taking a closer look at the psychological nature of the change in the individual’s life situation associated with this transition.

### 1.3. Shifting Opportunities and Changing Options: The Functionality of Goal Adjustment

The transition to retirement is typically associated with the fact that professional goals can no longer be achieved afterwards. This challenge is particularly significant when permanent goal intentions [30] are (perceived as) blocked. Permanent goal intentions (e.g., loyalty as a partner, democratic commitment, and fairness as a boss) are those that are important for personal identity and must be realized on an ongoing basis. It seems reasonable to assume that the individual’s capability to adapt goals to the changed opportunities should have a palliative effect on personal well‐being during the transition to retirement. As a consequence, the examination of the functionality of goal adjustment for this particular life transition seems promising.

Goal adjustment is shown to be a useful resource to maintain quality of life [17]. In particular, it can support or restore the feeling of leading a meaningful life [10], which in turn is an important predictor of many aspects of a successful life [31]. In the case of retirement, there is preliminary evidence that flexible goal adjustment (FGA) can be seen as a resource for maintaining psychological functioning [32]. There are several theoretical models that describe goal regulation processes as functional, particularly in older adults ([10, 11, 11, 12, 22, 23, 33, 34]; for comparison, e.g., [13]). In general, these approaches distinguish between an “assimilative” mode, in which relevant goals are maintained and tenaciously pursued even in the face of resistance and obstacles, and an “accommodative” mode [10], in which blocked goals are devalued and, in the best case, replaced by achievable goals. Although these processes of goal adjustment comprise a “family” of possible mechanisms [35], the conceptual core entails disengagement from a blocked goal [36] and engagement for a different, reachable one (see also [12]); models of goal adjustment, and, in particular, of goal disengagement, have been tested with respect to several life stages (mainly across adulthood) and with respect to a couple of goal blockages and threats [10, 11, 18]. However, the particular functionality of goal adjustment in relation to retirement as a sample case for life transition and change of goal achievement opportunities in later life has rarely been empirically investigated. In addition, the vast majority of studies that have investigated the functionality of goal adjustment in later life have not examined the (change of) patterns of goal importance directly, but rather the (self‐report of) individual disposition to adjust goals as a moderator.

### 1.4. The Current Study

The first aim of the present study was to assess individual differences in goal adjustment, first as an (self‐assessed) individual trait and, second, as a more state‐related difference (pre‐ vs. post‐retirement) with respect to (self‐reported) importance of professional goals. The second aim was to investigate the predictive value of goal adjustment for personal well‐being before versus after retirement.

In order to ensure a high self‐relevance of professional goals, the profession “pastor” was chosen. The educational path to this profession is chosen purposefully by the individual, and many of the associated activities (church services, weddings, funerals, etc.) are difficult to perform authentically without a high degree of personal commitment and beliefs. Moreover, almost all professional activities as a pastor constitute the central meaning of this particular profession and rely on self‐relation and self‐relevance (e.g., serving god, expressing faith, and supporting the community). The choice of becoming a pastor is often accompanied by the experience of being called to this profession [37–39]. It is plausible to assume that at least some of the professional goals that are reported by clergy (e.g., [40]), for example, helping others or serving the community, are permanent goal intentions or identity goals [41] that can never be conclusively achieved, but have to constantly be renewed in order to stay true to one’s identity (as a pastor or a servant of faith). This special constellation allows for the hypothesis that goal disengagement processes should be especially important for this population nearing retirement, since retirement from this profession is potentially associated with a blocking of several personally important (i.e., identity‐related) professional activities and, hence, goals. It is in the nature of identity goals that they can be (and must be) achieved via different means in different life phases. Thus, it is quite possible that while the overarching goal remains unchanged, the lower levels of the goal structure are adapted in a way to allow for the changed circumstances in retirement [6, 18].

For the current paper, we focus on the following hypotheses:

1. Retirement is associated with goal adjustment: in contrast to private goals, professional goals are rated as less important by participants in retirement than by working participants.
2. The individual preparedness for flexible goal disengagement predicts goal adjustment processes: retirees with an individual preparedness for goal adjustment rate professional goals, but not private goals, as less important than retirees with less flexibility.
3. Goal disengagement is functional:
  a. Participants with a higher preparedness for goal adjustment report higher well‐being and self‐esteem, especially in retirement.
  b. Retired participants who report less goal importance for professional goals report higher well‐being and self‐esteem.

## 2. Method

### 2.1. Design

This study followed a quasiexperimental cross‐sectional design with the between‐subjects factor retirement (before retirement vs. after retirement) and the within‐subject factor goal domain (private vs. professional).

### 2.2. Participants and Procedure

A cooperation with the local protestant church allowed us to invite pastors over 60 years old to participate. Our sample included working as well as retired participants. We also invited office staff to participate. We assumed that pastors—in contrast to office staff—would identify themselves more with their profession. However, the two groups were too different in size to compare (office staff members *n* = 31), thus only the pastors’ data are reported. Accordingly, our hypotheses concentrate on pastors only, since we would have expected a potential difference in goal adjustment for office staff members.

Assessment took place over four years (2020–2023; June to September of each year) with the possibility to participate each year. The study was conducted online using SoSci Survey [42]. On the study homepage, a data privacy statement as well as the informed consent information were available with the link to the study measures. All participants gave written informed consent and were treated in accordance with the Declaration of Helsinki, World Medical Association [43]. The study was approved by the local ethics board of the university (Ethikkommission des Fachbereichs 1, Universität Hildesheim, approval number 131).

For the current paper, we included only participants who filled out the complete questionnaire for the first time (cross‐sectional). The final sample consisted of 206 participants. The age range was from 62 to 80 years (*M*_age_ = 67.01, SD_age_ = 3.4). 67 participants identified as women and 139 as men. 46.1% were already retired. The number of years left until retirement ranged from 0 to 7 years. The number of years already spent in retirement ranged from 0 to 14 years. Figure 1 shows the distribution of retirees over different ages.

**Figure FIGURE 1:**
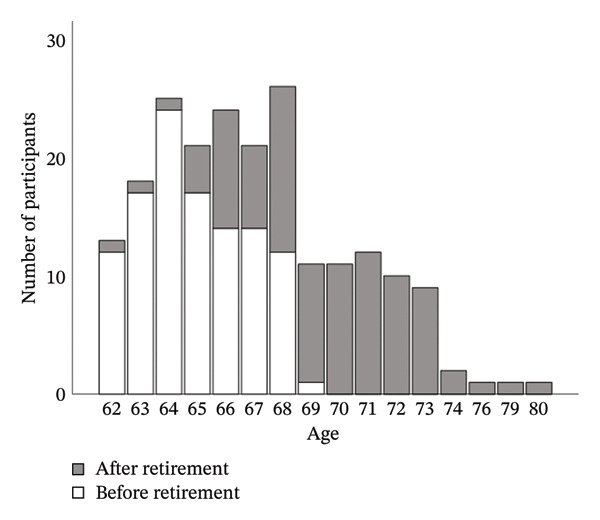
Distribution of retirees over different ages.

### 2.3. Measures

Only the measures relevant to the hypotheses are reported. They were part of a larger questionnaire which can be found here: https://osf.io/3thk5/?view_only%3d;e20e91d639684922b1d1b00f4df7309b. Additionally, links to three online reaction time experiments were provided (in 2020 and 2021). Due to low participation, this part of the study was not analysed further and will not be reported. All items were presented in German.

#### 2.3.1. Dispositional Goal Disengagement

The individual preparedness to disengage from goals was assessed with the Ten‐Flex scale derived from the two‐process model [34]. It consists of two subscales with 15 items each: the tenacious goal pursuit (TGP; sample item: “when faced with obstacles, I usually double my efforts.”) scale and the FGA (sample item: “I adapt quite easily to changes in plans or circumstances.”) scale. Participants rated their agreement on a 5‐point Likert scale from 1 = strongly disagree to 5 = strongly agree. A mean score was calculated both for TGP and FGA, respectively; both scales showed sufficient internal consistency (TGP: *α* = 0.72; FGA: *α* = 0.78).

#### 2.3.2. Goal Attachment: Importance of Personal and Professional Goals

Following Hamm et al. [44], the level of goal importance was assessed with a single item in four goal domains, respectively. Participants rated the personal current importance of these domains on a 15‐point scale. The question was phrased as follows: “In the following, different areas are presented in which people can pursue goals and invest time. Please rate how important each area is for you at the moment.” There were four domains: (1) private life/social contacts/leisure time, (2) personal development/health belonging to the private goal domains, (3) working with people/working in a team, and (4) professional development/professional qualifications belonging to the professional goal domains. The four domains were derived from a previous interview study [40]. The two private and two professional domains were combined, respectively, to represent mean goal importance for personal (*α* = 0.53, *r* = 0.36) and for professional goals (*α* = 0.62, *r* = 0.46).

#### 2.3.3. Self‐Esteem

Self‐esteem was assessed with a German version of the Rosenberg self‐esteem scale [45]. Participants rated their agreement on a 4‐point Likert scale. The 10 items showed a sufficient internal consistency (*α* = 0.81); a mean score of self‐esteem was calculated.

#### 2.3.4. Well‐Being

General well‐being was assessed with the WHO‐5 scale (German version, [46]). This scale represents the general well‐being in the past 2 weeks (example item “In the past 2 weeks, I was happy and in a good mood.”). Participants rated how often the statements were true on a 6‐point Likert scale from 1 = at no time to 6 = at all times. As the five items showed sufficient internal consistency (*α* = 0.83), they were combined to a mean of general well‐being.

#### 2.3.5. Subjective Health

As a control variable, we assessed subjective health with a one‐item question: “All in all, how would you rate your health status?”. Participants rated the item on a 10‐point Likert scale from 1 = very bad to 10 = very good.

#### 2.3.6. Expectations Regarding COVID‐19

Our data collection took place during the COVID‐19 pandemic. Starting in 2020, we included three exploratory questions regarding the expected influence of the pandemic on three areas of daily life. The items were phrased as follows: “Do you think the COVID pandemic will have long‐term consequences for you economically/health‐wise/socially?”; they were rated on a 5‐point Likert scale from 1 = no consequences to 5 = very big consequences.

### 2.4. Data Analysis

Since there were only few (mean = 2.6%) missing values in the overall data set, no missing data treatment was conducted. The data were analysed using IBM SPSS 29. A sensitivity analysis using G^∗^ Power 3.1 [47, 48] for *F*‐tests (analysis of variance (ANOVA) with fixed effects, between factors) as well as *t*‐tests (linear multiple regression and single regression coefficient) was calculated to determine which effect sizes can reliably be detected with the existing sample size of *N* = 206, an intended power of 0.95, and an alpha error of 0.05. For both analyses, medium to small effect sizes can be detected (*f* = 0.25 and *f* ^2^ = 0.05, respectively). The data were analysed based on the general linear model (GLM) using significance tests for correlations, regression models, and mean differences (ANOVA). The significance level was set at *α* = 0.05.

## 3. Results

Table 1 shows the Pearson correlations for all measures. The negative correlation between age and the importance of professional goals indicates the expected disengagement from professional goals in later life. The correlation between retirement and the importance of professional goals corresponds to hypothesis 1 and indicates that professional goals are rated as less important by participants in retirement than by working participants, in contrast to private goals for which no significant correlation with retirement was found. Furthermore, the correlations between FGA and self‐esteem and well‐being confirm earlier results with respect to the palliative function of (dispositional) goal adjustment.

**Table TABLE 1:** Pearson correlations for all measures.

	1	2	3	4	5	6	7	8
1. Age								
2. Retirement status	0.676^∗∗^							
3. TGP	0.012	−0.017						
4. FGA	−0.136	−0.053	0.036					
5. PRO‐GI	−0.289^∗∗^	−0.289^∗∗^	0.149^∗^	0.029				
6. PRIV‐GI	−0.047	0.133	−0.013	0.044	0.045			
7. Self‐esteem	−0.225^∗∗^	−0.159^∗^	0.241^∗∗^	0.415^∗∗^	0.129	0.182^∗∗^		
8. Well‐being	0.023	0.117	0.265^∗∗^	0.347^∗∗^	0.017	0.17^∗^	0.492^∗∗^	
9. Health	−0.071	0.051	0.173^∗^	0.214^∗∗^	0.164^∗^	0.054	0.427^∗∗^	0.499^∗∗^

*Note:* Retirement status, 1 = working and 2 = retired; PRO‐GI = professional goal importance; PRIV‐GI = private goal importance.

Abbreviations: FGA = flexible goal adjustment, TGP = tenacious goal pursuit.

^∗^ *p* < 0.05.

^∗∗^ *p* < 0.01.

*Retirement is associated with goal adjustment: In contrast to private goals, professional goals are rated as less important by participants in retirement than by working participants.*

To test hypothesis 1, an ANOVA with the between‐subjects factor retirement status (before vs. after) and the within‐subject factor goal domain (private vs. professional) was conducted over goal importance. Corresponding to the correlations found (see Table 1), a main effect goal domain, *F* (1, 204) = 190.05, *p* < 0.001, $\eta_{p}^{2}$ = 0.482, indicates that private goals were rated as more important (12.56 ± 1.76 SD) than professional goals (9.64 ± 2.84 SD). Confirming the hypothesis, there was also a significant interaction, *F* (1, 204) = 23.57, *p* < 0.001, $\eta_{p}^{2}$ = 0.104, showing that retired individuals rated professional goals as less important (8.76 ± 2.87 SD) than working individuals (10.4 ± 2.60 SD), in contrast to private goals which are rated as equally important by retired (12.81 ± 1.61 SD) and working individuals (12.34 ± 1.86 SD).

*The individual preparedness for flexible goal disengagement predicts goal adjustment processes: Retirees with an individual preparedness for goal adjustment rate professional goals, but not private goals, as less important than retirees with less flexibility.*

To test hypothesis 2, a regression model was used to predict the importance of professional goals with FGA, retirement status, and their interaction. The same model was also used to predict the importance of private goals. Consistent to the results from the Pearson correlations (Table 1), none of the predictors for professional or private goals reached significance (all *p*s > 0.17).

*Goal disengagement is functional: Participants with a higher preparedness for goal adjustment report higher well-being and self-esteem, especially in retirement.*

To test hypothesis 3a, two ANOVAs with the between‐subjects factor retirement status (before vs. after) and FGA (median‐split; high vs. low preparedness) were conducted with respect to well‐being and self‐esteem as dependent variables, respectively. For well‐being, a main effect of FGA, *F* (1, 202) = 19.88, *p* < 0.001, $\eta_{p}^{2}$ = 0.09, indicates that people with high FGA show higher well‐being (18.25 ± 3.30 SD) than people with low FGA (15.87 ± 4.47 SD), corresponding to the result of a positive correlation between FGA and well‐being (Table 1) and confirming our hypothesis. The main effect of retirement status just missed the criterion for significance (*p* = 0.059, $\eta_{p}^{2}$ = 0.018). Contrary to our hypothesis, the interaction (*p* = 0.788, $\eta_{p}^{2}$ = 0.00) was not significant.

For self‐esteem, a main effect of FGA, *F* (1, 201) = 30.68, *p* < 0.001, $\eta_{p}^{2}$ = 0.13, indicates that people with high FGA show higher self‐esteem (3.67 ± 0.27 SD) than people with low FGA (3.37 ± 0.47 SD), confirming our hypothesis. Additionally, the main effect of retirement status was significant (*F* (1, 201) = 5.22, *p* = 0.023, $\eta_{p}^{2}$ = 0.025), indicating higher self‐esteem before retirement (3.59 ± 0.36 SD) than after (3.45 ± 0.44 SD). Contrary to our hypothesis, the interaction was not significant (*p* = 0.193, $\eta_{p}^{2}$ = 0.008).

*Goal disengagement is functional: Retired participants who report less goal importance for professional goals report higher well-being and self-esteem.*

To test hypothesis 3b, two ANOVAs with the between‐subjects factor retirement status (before vs. after) and professional goal importance (median‐split; high vs. low) were conducted over well‐being and self‐esteem, respectively. Contrary to the hypothesis, none of the factors reached significance; for self‐esteem, retirement status (*p* = 0.063, $\eta_{p}^{2}$ = 0.017) and professional goal importance (*p* = 0.085, $\eta_{p}^{2}$ = 0.015) and their interaction were not significant (*p* = 0.942, $\eta_{p}^{2}$ = 0.000). For well‐being, there were also no significant effects (all *p*s > 0.116)^1^.

*Exploratory analyses: A more fine-grained analysis of timing*

Our sample contains participants who range from 0 to 14 years to or from the point of retirement. Since earlier studies have found expectation effects for adjustment processes before a work transition [49], we assumed that a more fine‐grained distinction of time before and after retirement could add a better understanding to our results. Thus, we ran the same analyses as above, replacing the variable retirement status with distance from retirement (DfR). This new variable consisted of five groups: > 2 years prior to retirement (*n* = 66), < 2 years prior to retirement (*n* = 42), year of retirement (*n* = 12), < 2 years after retirement (*n* = 42), and > 2 years after retirement (*n* = 44). With the exception of the middle group, group sizes were comparable enough to allow for analysis.

The results supported hypothesis 1. In an ANOVA with DfR (between‐subjects) and goal domain (within‐subject) as factors, we found a significant main effect goal domain, *F* (1, 201) = 156.25, *p* < 0.001, $\eta_{p}^{2}$ = 0.437, and an interaction between goal domain and DfR, *F* (1, 201) = 6.48, *p* < 0.001, $\eta_{p}^{2}$ = 0.114.

For hypothesis 2, DfR did not predict professional (*p* = 0.681, regression model *R* ^2^ = 0.080) or private (*p* = 0.453, *R* ^2^ = 0.016) goal importance in the regression model with FGA and their interaction.

The prior results for hypothesis 3a were partially confirmed by an ANOVA with DfR and FGA (median‐split) as between‐subjects factors, with DfR showing a significant main effect for self‐esteem (*F* (1, 195) = 3.01, *p* = 0.019, $\eta_{p}^{2}$ = 0.058), but not well‐being (*p* = 0.180, $\eta_{p}^{2}$ = 0.031). For hypothesis 3b, an ANOVA with the between‐subjects factors DfR and professional goal importance (median‐split) was conducted. DfR showed a significant main effect for self‐esteem, *F* (1, 195) = 3.04, *p* = 0.018, $\eta_{p}^{2}$ = 0.059, and a main effect for well‐being which just missed the criterion for significance, *F* (1, 196) = 2.4, *p* = 0.051, $\eta_{p}^{2}$ = 0.047. The interactions were not significant (*p* = 0.835 for self‐esteem, *p* = 0.673 for well‐being). Post hoc tests revealed that all groups differ significantly from the group “> 2 years after retirement” (except for “year of retirement,” *p* = 0.332), indicating lower self‐esteem more than 2 years after retirement (3.32 ± 0.51 SD) than before (“< 2 years after retirement” 3.58 ± 0.32 SD, *p* = 0.030; “< 2 years prior to retirement” 3.64 ± 0.28 SD, *p* = 0.003; “> 2 years prior to retirement” 3.54 ± 0.41, *p* = 0.044).

For hypothesis 1, an even finer look at the years prior and after retirement seemed promising. Thus, we replaced the variable “DfR” with “distance from retirement in 9 groups” (DfR9). This new variable consisted of nine groups: “> 3 years prior to retirement” (*n* = 47), “3 years prior to retirement” (*n* = 19), “2 years prior to retirement” (*n* = 17), “1 year prior to retirement” (*n* = 22), “year of retirement” (*n* = 22), “1 year after retirement” (*n* = 17), “2 years after retirement” (*n* = 18), “3 years after retirement” (*n* = 15), and “> 3 years after retirement” (*n* = 29). In an ANOVA with DfR9 (between‐subjects) and goal domain (within‐subject) as factors, we again found a significant main effect goal domain, *F* (1, 197) = 174.30, *p* < 0.001, $\eta_{p}^{2}$ = 0.469, as well as an interaction between goal domain and DfR9, *F* (8, 197) = 3.61, *p* < 0.001, $\eta_{p}^{2}$ = 0.128, indicating different relations between professional and private goal importance in the groups around the year of retirement, respectively, thus confirming hypothesis 1 even with the between‐subjects factor split into nine groups. The means for each group are shown in Figure 2. The professional goal importance was lowest for the group with longest post‐retirement (> 3 years), possibly showing that devaluating the goals either takes some time or is not immediately necessary.

**Figure FIGURE 2:**
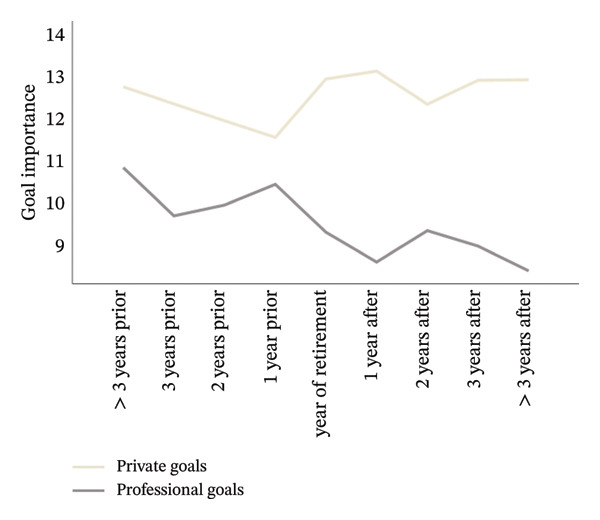
Mean goal importance for private and professional goals in each group.

### 3.1. Exploratory Analyses: The Influence of COVID‐19

Table 2 shows the correlations of the three COVID‐19‐related items with our central variables. While expected economic consequences correlate negatively with retirement status, expected social and health consequences show negative correlations with well‐being as well as FGA.

**Table TABLE 2:** Pearson correlations for all measures with COVID‐19 items.

	COVID‐19 economic	COVID‐19 health	COVID‐19 social
1. Age	−0.117	−0.031	0.019
2. Retirement status	0.182^∗∗^	0.046	−0.048
3. TGP	−0.001	−0.141^∗^	−0.055
4. FGA	−0.054	−0.217^∗∗^	−0.175^∗^
5. PRO‐GI	0.101	−0.054	0.032
6. PRIV‐GI	−0.086	−0.098	−0.087
7. Self‐esteem	−0.121	−0.220^∗∗^	−0.119
8. Well‐being	−0.082	−0.287^∗∗^	−0.234^∗∗^
9. Health	−0.012	−0.312^∗∗^	−0.177^∗^

*Note:* Retirement status, 1 = working and 2 = retired; PRO‐GI = professional goal importance; PRIV‐GI = private goal importance.

Abbreviations: FGA = flexible goal adjustment, TGP = tenacious goal pursuit.

^∗^ *p* < 0.05.

^∗∗^ *p* < 0.01.

## 4. Discussion

The purpose of the present study was to examine whether the transition to retirement initiates goal adjustments as a coping response for a professional group in which a high degree of personal identification with the professional activity can be expected (pastors) and whether these adjustments are functional for maintaining well‐being.

In line with our hypothesis, the importance of professional goals was lower for retired pastors than for their working colleagues, while the importance of private goals did not differ between working and retired pastors. This difference in goal attachment supports the assumption that goal disengagement plays a role in the adjustment process after retirement.

Although the cross‐sectional design of the present study limits the interpretation of between‐subject differences as indicators of dynamics of adjustment (e.g., cohort effects cannot be excluded), it can be argued that since recruitment for all age groups was absolutely identical, and the differences with respect to birth cohorts (about 15 years) are relatively small, the claim of a cohort effect (and hence an artificial effect) carries the burden of proof. Actually, since the sample examined here was recruited over several years, cohort effects are unlikely as an alternative explanation for the differences in goal importance. At the same time, the fact that the data have been collected over four subsequent years reduces the plausibility of a period effect (or an interaction with a cohort effect). If alternative explanations (such as cohort effects or similar) are not plausible, the individual differences in the importance of goals identified here are, after all, a necessary condition for the assumptions of goal disengagement theories to be valid. In this sense, the available data test the predictions of goal adjustment theories.

Regarding the question of functionality, our findings show that individual preparedness for goal adjustment is associated with self‐esteem and well‐being, both before and after retirement. Contrary to our expectations, the reported importance of professional goals itself did not predict well‐being or self‐esteem. It might be plausible to assume that well‐being and self‐esteem do not directly depend on, nor even are susceptible to, importance of a selection of professional or private goals (both facets of quality of life may rather depend on the “amount” of attainment with respect to these goals).

In addition to testing these hypotheses, the present study extends earlier research with respect to the assessment of goal adjustment. Firstly, most studies, within the framework of the two‐process model and related approaches, only indirectly measure accommodative processes and their effectiveness via the buffering effect of (self‐reported) individual disposition to respond accommodatively. Only few studies on the two‐process model have so far examined actual changes in goal importance (for a recent exception, see [50]) and only in relation to very short time periods (less than an hour: [51, 52]). Secondly, the existing studies have so far only examined more serious critical life events or developmental transitions in exceptional cases. Although the transition to retirement has been discussed occasionally as a prototypical occasion for accommodative regulation [53], to our knowledge, no specific study has been conducted on this topic to date. Thirdly, there is a methodological aspect: even though the present findings do not yet constitute a direct test of accommodative change because they do not present longitudinal data, the current study avoids self‐reports regarding the accommodative process. Instead, in addition to a dispositional self‐report scale of the individual’s preparedness for goal adjustment, individual differences with respect to self‐reported goal importance were used as an indicator of current goal attachment to private and professional goals. Although the limits of self‐report data in general limit the interpretation of results with respect to reliability and, hence, internal validity, it can be argued that with respect to goal importance, self‐report (given it can be seen as frankly given) can be seen as valid. Actually, goal disengagement approaches (provided they address not only behavioral but also affective‐motivational goal adjustments) would predict precisely the adjustment of the subjective importance of goals. In this respect, the use of self‐report data in this respect tests a central thesis of these approaches. Accordingly, the indirect assessment of adjustment of (directly assessed) goal importance might be considered as a (state‐oriented) validation of the (perhaps less reliably assessed by self‐reports) general preparedness of goal adjustment.

### 4.1. The Relationship Between Goal Attachment and the Preparedness for Goal Adjustment

Hypothesis 2 assumed that a high preparedness for goal adjustment would be associated with low goal attachment to unattainable goals, indicating a successful disengagement (e.g., [10]). However, the individual preparedness for flexible goal adjustment did not predict the importance of professional goals in our sample. One possible explanation for this finding could be that the wording of the items asking about the professional goal domains might have been misleading. While the goal domains were selected especially for pastors (in a prestudy; [40]), it could be the case that working with people/working in a team and professional development/professional qualifications was understood as something one can achieve in retirement as well, thus undermining the concept of professional goals. However, the difference between importance of professional goals, but not private goals, for working participants and retirees can be interpreted as a validation of the items. Perhaps the transition to private life is less “direct” for pastors than in other professions. In fact, pastors often report that they continue to take on professional duties on a temporary basis for some time after retiring (e.g., weddings, church services, or funerals). This is supported by the fact that the decline in the importance of professional goals was most pronounced among the group whose retirement was furthest in the past.

### 4.2. Goal Adjustment as a Part of Retirement Planning

From a practical perspective, the present study extends the literature by investigating a critical life event (retirement) that can be anticipated individually. A predictable retirement is experienced more positively [19], and preretirement planning, including psychological preparation, is associated with higher life satisfaction and less psychological distress after retirement [54]. While these processes are often conceptualized as deliberate preparations for retirement, they can also include nondeliberate preplanning processes which prepare, or facilitate, conscious decision making [55]. These processes could include preparations of adaptations in goal structures (as predicted by goal adjustment theories) while approaching the retirement date, as our results show a growing difference in goal importance for professional goals compared to private goals already around 2 years before retirement.

On an individual level, goal adjustment could be part of these processes preparing for retirement. If goals that cannot be pursued following retirement are devalued, those goals that maintain their importance (e.g., family, leisure, and social engagement) gain dominance, thereby allowing and guiding individual action. In line with the notion that goals that are no longer attainable are devalued, our results show a difference in priorities regarding private and professional goal domains in retirement in contrast to working life. On a social level, it might be worth considering the significance of plurality of goals and, in particular, the relativization of professional goals, even in the case of professions that are so strongly driven by identity goals.

### 4.3. Limitations

The present study focused on the potential palliative effect of goal disengagement during retirement. In fact, our findings contribute to the growing evidence that retirement is not always associated with declines in well‐being, but can improve quality of life [25, 27, 56, 57]. We observed significant differences in self‐esteem before and after retirement, which were primarily driven by the group that had been retired for more than 2 years. Further research is needed, particularly with samples of individuals who have been retired longer or experience a more pronounced decline in well‐being directly after retirement.

Our selection of a profession with assumed high self‐relevance of professional goals might also contribute to the described result patterns. In our sample, participants had been working in their current position for the protestant church up to 42 years, with a mode of 34 years, confirming the expected commitment to their chosen profession. Additionally, 47.1% are still active in church services after the end of their formal occupation (as substitutes for absent pastors, in voluntary roles, etc.). This is in line with recent discussions regarding retirement processes. Lassen and Vrangbæk [58] argue that the transition to retirement has undergone significant changes in the last years. Beyond the strict dichotomy of working versus retired, there may be increasingly longer, and more diverse, periods of blended states. The distinction made here between professional activity and retirement is therefore probably too crude for this professional group in particular. However, the weakened distinction between working and retired individuals should have a conservative influence on the significance of differences investigated in our studies. Given this, any differences between working and retired individuals in our study should be considered more indicative of changes associated with the transition than if there was a clear distinction between working status and retired status in our sample. Thus, the significant difference in the importance of professional goals for retired pastors in contrast to still working participants shows that even within this specific population, retirement seems to support an adaptation of goal structures regarding professional areas.

Even though the study was planned as a longitudinal assessment with both direct (questionnaire) and indirect (reaction time based) measures, low (repeated) participation rates limited the analysis to a cross‐sectional design. Thus, the limitations of cross‐sectional findings with respect to causal interpretation must be taken into account. As mentioned before, the present study is not able to assess the individual goal adjustment process, but rather interindividual differences in goal importance before and after retirement. However, this does not necessarily render the present results meaningless; rather, it can be argued (as discussed before) that the results have their merit in approving and supporting (beyond replication) earlier findings.

Actually, the sample is self‐selective. It is conceivable that participation in the survey was influenced, among other things, by the extent to which the transition to retirement age was experienced as particularly stressful. Longitudinal data would be able to partially compensate also for this problem. However, since no indications of any differences in recruitment selectivity between active and retired pastors are visible, the present cross‐sectional pattern might be seen as a first indicator of an actual process of goal adjustment. Another characteristic from our self‐selected sample is the distribution of gender, with 67 women and 139 men. A test for gender differences revealed statistically significant differences (all *p*s < 0.02) in our sample for age (women having a lower mean age than men), retirement status (most women still work while more men are retired), importance of professional goals (women rate professional goals as more important than men do), and self‐esteem (women having higher mean self‐esteem than men). However, these differences seem trivial given that self‐esteem, the importance of professional goals, and age are all correlated with the retirement status, and most women (70.15%) in our sample still work. Moreover, the contrast of group sizes requires caution when interpreting these results. It should also be noted that the decision to analyze portions of the data using median splits conferred the advantages of producing equally sized groups and facilitated the interpretation of results. However, this approach entailed the drawback of reducing variance and potentially discarding information inherent in the original data. With respect to the fact that the present findings are based on self‐report data, it is noteworthy that (change of) subjective goal attachment (as assessed by goal importance in the present study) is directly entailed in all models of goal adjustment as they precisely predict an adjustment in goal attachments. Subject to longitudinal confirmation, the evidence presented here can therefore be seen as more direct evidence of the process of goal adjustment as a consequence of retirement. In contrast, the self‐report assessment of the individual’s preparedness for goal adjustment (which is present in the majority of studies of goal adjustment; [36]) is to be seen as a mere proxy for processes of adjustment.

### 4.4. Practical Implications

Our results show, firstly, that most retirees value professional goals less than working participants and, secondly, that the preparedness for goal adjustment is a predictor for well‐being and self‐esteem in retirement. If the readjustment of subjective goal valences can be prepared or facilitated, then this could help to ease both the transition into retirement and the general loss of options for pursuing professional goals that accumulate with age. Accordingly, helping individuals emphasize and possibly expand personal goals outside the area of professional activities could be a worthwhile objective for employers, therapists, insurers, and even government programs. This might be supported by encouraging social debates on the possible benefits of goal disengagement [36]. Certainly, these benefits emerge for other professional activities as well. In a sense, the present studies testing the adjustment of professional goals with pastors can be considered a conservative test: if even pastors are able to disengage from their professional goals (that are supposed to be a constitutive part of their personal identities, as it were), other professions should be able to adjust their goals as well, perhaps even more so.

## 5. Conclusion

Although the hypotheses were only partially supported by the available data, the findings presented provide indications that, at least for the occupational group examined here, goal adjustments are made after retirement. While these findings require longitudinal verification for a variety of occupational groups, the present results indicate that more extensive follow‐up studies would be promising. One practical implication, given that goal adjustments prove functional for coping with a foreseeable blockage of important goals, might be to implement preventive measures focusing on goal adjustment in preparation for retirement.

## Funding

Open access funding was enabled and organized by Projekt DEAL. We acknowledge financial support by Stiftung Universität Hildesheim.

## Conflicts of Interest

The authors declare no conflicts of interest.

## Endnotes

^1^Since the variable subjective health has significant correlations with our dependent variables self‐esteem and well‐being, we tested for hypotheses 3a and 3b with an additional ANCOVA. While subjective health is a significant factor in all analyses, the result pattern remains the same except for well‐being: when controlling for health, the factor retirement status has a significant effect on it (H3a: *F* (1, 193), *p* = 0.004; H3b: *F* (1, 193), *p* = 0.009).

## Data Availability

Data and codebook of this study can be found here: https://osf.io/3thk5/?view_only%3d;e20e91d639684922b1d1b00f4df7309b.
